# Phenotypic and genetic variation in phosphorus-deficiency-tolerance traits in Chinese wheat landraces

**DOI:** 10.1186/s12870-020-02492-3

**Published:** 2020-07-13

**Authors:** Yu Lin, Guangdeng Chen, Haiyan Hu, Xilan Yang, Zhengli Zhang, Xiaojun Jiang, Fangkun Wu, Haoran Shi, Qing Wang, Kunyu Zhou, Caixia Li, Jian Ma, Youliang Zheng, Yuming Wei, Yaxi Liu

**Affiliations:** 1 Triticeae Research Institute, Sichuan Agricultural University, Wenjiang, Chengdu, 611130 China; 2 College of resources, Sichuan Agricultural University, Wenjiang, Chengdu, 611130 China; 3 School of Life Science and Technology, Henan Institute of Science and Technology, Xinxiang, 453003 Henan China; 4 State Key Laboratory of Crop Gene Exploration and Utilization in Southwest China, Wenjiang, Chengdu, 611130 China

**Keywords:** Abiotic stress, Association analysis, Compressed mixed linear model, Genetic variation, Phosphorus-deficiency tolerance, *Triticum aestivum* L, Wheat landrace, Seedling stage, QTL, SNP

## Abstract

**Background:**

Phosphorus deficiency is a major limiting factors for affecting crop production globally. To understand the genetic variation of phosphorus-deficiency-tolerance, a total of 15 seedling traits were evaluated among 707 Chinese wheat landraces under application of phosphorus (AP) and non-application of phosphorus (NP). A total of 18,594 single-nucleotide polymorphisms and 38,678 diversity arrays technology sequencing markers were used to detect marker-trait associations under AP and NP.

**Results:**

Top ten genotypes with extremely tolerance and bottommost ten genotypes with extremely sensitivity were selected from 707 Chinese wheat landraces for future breeding and genetic analysis. A total of 55 significant markers (81 marker-trait associations) for 13 traits by both CMLM and SUPER method. These were distributed on chromosomes 1A, 1B, 2A, 2B, 2D, 3A, 4B, 5A, 5B, 6A, 6B, 6D, 7A and 7B. Considering the linkage disequilibrium decay distance, 25 and 12 quantitative trait loci (QTL) were detected under AP and NP, respectively (9 QTL were specific to NP).

**Conclusions:**

The extremely tolerant landraces could be used for breeding phosphorus-deficiency-tolerant cultivars. The QTL could be useful in wheat breeding through marker-assisted selection. Our findings provide new insight into the genetic analysis of P-deficiency-tolerance, and will be helpful for breeding P-deficiency-tolerant cultivars.

## Background

Phosphorus (P) is an essential macronutrient for plant growth, and yet it is a major limiting factor for crop production globally [1, 2]. Although total P in soil is abundant, it is largely unavailable for uptake by plants. Phosphorus readily forms complexes with metal cations, and microorganisms in the soil convert phosphate P into organic compounds [3–5], thus making P unavailable in soils. Phosphorus fertilizer is commonly used to overcome this problem. However, applying large quantities of fertilizer causes environmental concerns about fertilizers leaching into water systems, and accelerates the exhaustion of nonrenewable phosphate resources [6, 7]. It has been estimated that the global P resources will be exhausted by the end of this century [2, 8]. Thus, it is important to breed P-deficiency-tolerant varieties for sustainable agriculture.

Wheat (*Triticum aestivum* L.) is among the earliest domesticated crop plants, being cultivated 10,000 years ago in the pre-pottery Neolithic Near East Fertile Crescent [9, 10]. Currently, wheat is the most widely cultivated food crop, contributing about a 20% of the calories consumed by humans. P is an important limiting nutrient for wheat growth and development. Wheat yield has been severely limited by P deficiency globally [11, 12]. Hence, development of P-deficiency-tolerance wheat is critical. Landrace genotypes are an important resource for wheat improvement. Chinese wheat landraces have been shown to be enriched with genes and alleles that are tolerant or resistant to abiotic and biotic stress [13–16]. Therefore, screening tolerant genotypes and understanding the genetic basis of P-deficiency tolerance in Chinese wheat landraces could provide important insights for the breeding of tolerant wheat cultivars.

The genetic control of P-deficiency-tolerance traits has been investigated extensively using linkage mapping of bi-parental wheat populations. Quantitative trait loci (QTL) for P-deficiency-tolerance traits have been successfully identified using this approach [17–20]. An alternative method, which may have greater potential for improving P-deficiency tolerance, is to identify the allelic variations due to divergent selection pressures, within a large genetic diversity panel. Through genome-wide association studies (GWAS), such natural allelic variations for P-deficiency-tolerance have been identified in *Arabidopsis* [21], *Aegilops tauschii* [22], soybean [23], but not yet in wheat. Thus, our objective was to identify such allelic variations in wheat through GWAS. These findings will provide new insights for the improvement of P-deficiency tolerance.

Phosphorus nutrition during the early growing stage is critical for wheat final yield. P deficiency during the early growing stage causes large reductions in tiller development and head formation, and plants cannot recover from this, even if sufficient P is later supplied [24, 25]. Therefore, it is important to understand the genetic control of P-deficiency-tolerance during the seedling stage, and to develop P-deficiency-tolerant cultivars. In this study, we first examined phenotypic variation in 15 P-deficiency-tolerant traits in a core of 707 wheat landraces at seedling stage. We then evaluated the P-deficiency-tolerance of these landraces to identify suitable wheat germplasm for future breeding of tolerant wheat cultivars. Finally, we performed a GWAS using 57,272 polymorphism markers to identify marker–trait associations (MTAs).

## Results

### Phenotypic variation

To evaluate variation in the phenotypic response to P deficiency, 707 wheat landraces were grown under both application of phosphorus (AP) and non-application of phosphorus (NP). Fifteen traits were evaluated to determine the effect of P deficiency and their genetic variation at the seedling stage. There was significant variation among genotypes for all traits (*p* < 0.001; ANOVA; Table 1). The P treatment had highly significant effects on all traits (*p* < 0.001; Table 1). Phosphorus deficiency had negative effects on almost all traits, except for the dry root–shoot ratio (DRS), fresh root–shoot ratio (FRS), and root diameter (RD). The coefficients of variation for the traits were 1.22 to 49.09% under AP, and 1.21 to 48.40% under NP (Table 2).

**Table 1:** Variance analysis for the tested traits under application of phosphorus (AP) and non-application of phosphorus (NP)

Variables	Type III sum of squares		Mean Squares		F value		Significance	
	Genotype	Treatment	Genotype	Treatment	Genotype	Treatment	Genotype	Treatment
df	706	1	706	1	706	1	706	1
DRS	8.85	9.08	0.012	9.08	3.17	2292.51	***	***
FRS	52.14	83.31	0.07	83.31	3.97	4478.5	***	***
RD	2.65	0.47	0.0038	0.47	1.71	212.72	***	***
RDW	0.12	0.012	0.00016	0.012	6.40	485.66	***	***
RF	212,855,532.7	133,653,108.3	301,495.1	133,653,108.3	7.33	3247.4	***	***
RFW	33.06	21.14	0.048	21.14	6.29	2838.74	***	***
RL	61,471.86	18,706.07	87.07	18,706.07	3.88	834.46	***	***
RSA	234,356.7	153,275.36	331.95	153,275.36	5.94	2744.28	***	***
RT	271,319,964.8	56,198,030.7	384,305.9	56,198,030.7	6.44	941.31	***	***
RV	18.54	10.56	0.03	10.56	5.92	2380.57	***	***
SDW	0.94	0.77	0.0013	0.77	7.09	4089.51	***	***
SFW	47.14	165.52	0.068	165.52	3.70	9165.92	***	***
SL	52,187.5	100,420.59	73.92	100,420.59	3.78	5135.41	***	***
TL	145,012.95	205,857.11	205.4	205,857.11	3.15	3156.8	***	***
TRL	21,672,647.14	14,211,126.91	30,697.8	14,211,126.91	4.72	2185.79	***	***

*** represent significance level of *p* < 0.001

**Table 2:** Analysis of basic parameters and heritability for the tested traits under application of phosphorus (AP) and non-application of phosphorus (NP)

Traits	Range		Mean		SD		CV (%)		Heritability		Shannon-Weaver diversity index	
	AP	NP	AP	NP	AP	NP	AP	NP	AP	NP	AP	NP
DRS	0.24–0.45	0.32–0.50	0.30	0.40	0.032	0.03	10.67	7.50	0.57	0.56	0.81	0.82
FRS	0.39–0.83	0.59–1.18	0.56	0.84	0.07	0.09	12.50	10.71	0.62	0.62	0.86	0.81
RD	0.35–0.38	0.37–0.40	0.36	0.38	0.0044	0.0046	1.22	1.21	0.16	0.15	0.84	0.78
RDW	0.012–0.044	0.012–0.030	0.022	0.019	0.0048	0.0032	21.82	16.84	0.76	0.70	0.81	0.83
RF	313.74–2975.52	279.25–826.76	764.83	408.98	290.13	82.96	37.93	20.28	0.85	0.56	0.63	0.74
RFW	0.16–0.94	0.16–0.39	0.38	0.24	0.11	0.04	28.95	16.67	0.81	0.57	0.79	0.81
RL	16.19–32.61	13.66–28.69	24.23	20.03	2.77	2.43	11.43	12.13	0.62	0.58	0.86	0.83
RSA	16.24–84.95	15.62–35.31	33.95	21.88	9.00	2.90	26.51	13.25	0.81	0.53	0.74	0.80
RT	234.05–2701.40	148.33–1385.13	528.45	297.12	259.39	143.82	49.09	48.40	0.74	0.77	0.28	0.28
RV	0.15–0.68	0.13–0.35	0.31	0.21	0.075	0.03	24.19	14.29	0.78	0.64	0.79	0.83
SDW	0.043–0.16	0.031–0.079	0.075	0.048	0.017	0.0082	22.67	17.08	0.79	0.74	0.79	0.81
SFW	0.40–1.32	0.24–0.42	0.68	0.29	0.12	0.02	17.65	6.90	0.70	0.37	0.78	0.81
SL	29.90–46.76	21.94–35.66	38.59	28.86	2.65	2.29	6.87	7.93	0.59	0.63	0.85	0.87
TL	50.64–78.13	39.66–58.36	62.82	48.88	4.25	3.16	6.77	6.46	0.56	0.52	0.82	0.81
TRL	151.22–835.66	148.46–279.27	305.43	189.34	84.33	21.81	27.61	11.52	0.78	0.40	0.72	0.80

The broad-sense heritability (*H*^*2*^) was moderate to high for all traits except RD. heritability varied from 0.16 for RD to 0.85 for root forks (RF) under AP, and from 0.15 for RD to 0.77 for root tips (RT) under NP (Table 2). RD showed low heritability under both conditions. The Shannon–Weaver diversity index (*H′*), which shows the diversity of each trait, was 0.28 to 0.86 under AP and 0.28 to 0.87 under NP; it reflected moderate to high diversity for the traits, except for RT (Table 2).

### Correlation analysis

Under AP, correlation coefficients ranged from 0.018 to 0.976 (Table 3; correlations under AP are shown below the diagonal). With the exception of DRS, FRS, and RD, the correlations between all pairs of the 15 traits were significantly positive. DRS was significantly negatively correlated with shoot dry weight (SDW), shoot fresh weight (SFW), shoot length (SL), and total length of shoot and root (TL). DRS was significantly positively correlated with the other traits, and was not significantly correlated with RD, RF, and RT. FRS was significantly negatively correlated with SL, and significantly positively correlated with the other traits. RD was significant positively correlated with FRS, and significantly negatively correlated with the other traits, except for DRS and root volume (RV).

**Table 3:** Correlation coefficients among all tested traits under application of phosphorus (AP) and non-application of phosphorus (NP)

	TL	RL	SL	SFW	RFW	SDW	RDW	FRS	DRS	TRL	RSA	RD	RV	RT	RF
TL	1	.819**	.744**	.624**	.650**	.416**	.525**	.192**	.203**	.690**	.674**	−.365**	.584**	.255**	.459**
RL	.847**	1	.226**	.317**	.532**	.227**	.420**	.425**	.364**	.599**	.565**	−.397**	.462**	.282**	.270**
SL	.849**	.438**	1	.690**	.484**	.442**	.403**	−.168**	−.078*	.473**	.486**	−.156**	.453**	.104**	.463**
SFW	.692**	.618**	.555**	1	.762**	.786**	.707**	−.112**	−0.073	.662**	.731**	−0.038	.742**	.309**	.706**
RFW	.634**	.687**	.391**	.869**	1	.771**	.917**	.526**	.330**	.834**	.920**	−.106**	.932**	.501**	.756**
SDW	.656**	.610**	.503**	.962**	.903**	1	.827**	.165**	−.165**	.587**	.696**	0.065	.761**	.469**	.650**
RDW	.565**	.621**	.338**	.728**	.889**	.791**	1	.474**	.397**	.776**	.871**	−0.062	.899**	.536**	.761**
FRS	.169**	.390**	−.101**	.163**	.603**	.292**	.637**	1	.611**	.394**	.430**	−.134**	.428**	.285**	.214**
DRS	−.097**	.092*	−.256**	−.257**	.094*	−.194**	.415**	.653**	1	.366**	.355**	−.209**	.306**	.128**	.233**
TRL	.672**	.717**	.423**	.817**	.911**	.854**	.843**	.497**	.084*	1	.965**	−.494**	.826**	.590**	.859**
RSA	.645**	.703**	.392**	.851**	.965**	.889**	.879**	.556**	.097**	.976**	1	−.280**	.944**	.616**	.868**
RD	−.389**	−.347**	−.312**	−.150**	−.087*	−.150**	−.132**	.081*	0.040	−.390**	−.200**	1	0.033	−.171**	−.286**
RV	.572**	.639**	.332**	.830**	.962**	.868**	.864**	.593**	.114**	.888**	.966**	0.033	1	.583**	.794**
RT	.453**	.525**	.244**	.621**	.648**	.648**	.582**	.258**	−0.043	.796**	.765**	−.289**	.683**	1	.644**
RF	.532**	.538**	.364**	.809**	.860**	.858**	.782**	.413**	−0.018	.931**	.924**	−.284**	.858**	.825**	1

Using ‘1’ as diagonal line, the correlation of all tested traits under AP and NP are below and above the diagonal line, respectively;* and ** represent significance level of *P* < 0.05 and *P* < 0.01, respectively

Under NP, the correlation coefficients ranged from 0.033 to 0.965 (Table 3; correlations under NP are shown above the diagonal). With the exception of DRS, FRS, and RD, all pairs traits were significantly positively correlated. DRS was significantly negatively correlated with RD, SDW, and SL, and significantly positively correlated with the others except for SFW. FRS was significantly negatively correlated with RD, SFW, and SL, and significantly positively correlated with the others. RD was significantly negatively correlated with all traits except root dry weight (RDW), RV, SDW, and SFW.

Under both AP and NP, root surface area (RSA) and total root length (TRL) had the highest correlations. With the exception of DRS, FRS, and RD, all pairs of correlations were significantly positive. Among the six root morphological traits (RF, root length (RL), RSA, RT, RV, and TRL), the correlations were moderately to highly positive. The other root morphological trait, RD, was negatively correlated with RF, RL, RSA, RT, and TRL under both conditions.

### Principal component (PC) analysis

The PC analysis were performed using the relative trait values. The cumulative amount of phenotypic variation explained (PVE) by the first three PCs was 82.98% (Table 4). PC1 explained 57.84% of the phenotypic variation. With the exception of DRS, FRS, and RD, all of the other traits were important factors within the characteristic vector of PC1. PC1 represented plant biomass and root architecture, and can thus be defined as the biomass and root factor. PC2 (eigenvalue 2.53) explained 16.84% of the phenotypic variation. DRS and FRS, which influence P absorption in shoots and roots under P-deficiency, were important factors within the characteristic vector of PC2. PC2 can thus be defined as the root–shoot ratio factor. The relative values of TL, SL, SFW, SDW, and RT for PC2 were negative, indicating that an increase in the root–shoot ratio will reduce TL, SL, SFW, SDW, and RT. PC3 (eigenvalue 1.24), for which RD was the only important factor, explained 8.29% of the phenotypic variation. The relative values of TL, RL, SL, FRS, DRS, TRL, and RT were negative for PC3, indicating that an increase in RD will correspond to a reduction in TL, RL, SL, FRS, DRS, TRL, and RT.

**Table 4:** Principal component analysis (PCA) of all tested traits

	Trait	PC1	PC2	PC3
Characteristic vector	DRS	0.06	0.90	−0.15
	FRS	0.09	0.89	−0.09
	RD	−0.45	0.01	0.75
	RDW	0.80	0.39	0.08
	RF	0.91	0.09	0.10
	RFW	0.92	0.23	0.21
	RL	0.78	0.00	−0.28
	RSA	0.96	0.20	0.11
	RT	0.54	−0.18	−0.44
	RV	0.90	0.23	0.3
	SDW	0.78	−0.44	0.22
	SFW	0.86	−0.31	0.29
	SL	0.73	−0.44	−0.15
	TL	0.86	−0.27	−0.25
	TRL	0.93	0.14	−0.02
Eigenvalues		8.68	2.53	1.24
Contribution%		57.84	16.84	8.29
Cumulative contribution%		57.84	74.69	82.98

### Screening for wheat tolerant genotypes

**Fig. 1:**
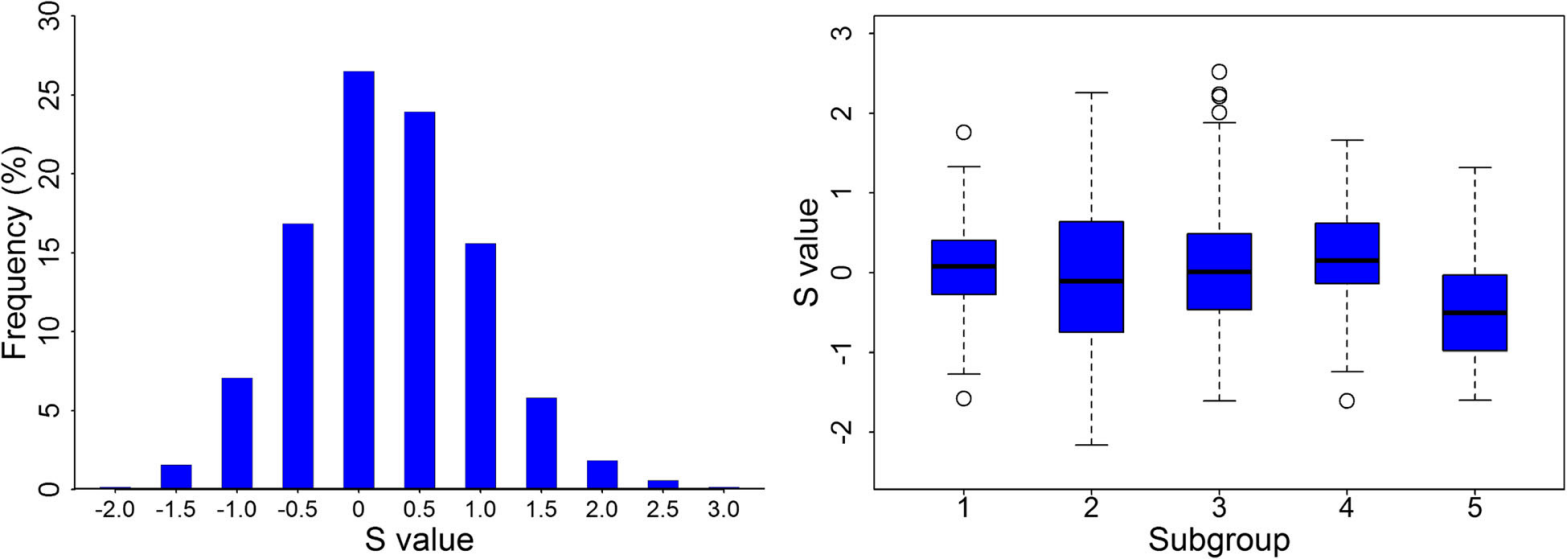
Distribution histograph and box plots for S value

Using a weighting method [26], the synthesis value (*S* value) was calculated to evaluate wheat tolerance to P deficiency, and the P-deficiency tolerance index (PDTI) for each trait was calculated. The accessions with extremely high or low *S* values are listed in Additional file 1: Table S1. A high *S* value indicates high tolerance. The wheat landraces were classified into three groups, ranging from − 2.16 (accession AS661384) to 2.52 (accession AS661809) (Fig. 1). There were 173 accessions in the first group (*S* ≥ 0.5; classified as high tolerance). Group 2 (− 0.5 ≤ *S* < 0.5; intermediate tolerance) included 353 accessions. The remaining accessions (*S* < 0.5) were classified as sensitive. Accessions with higher *S* values also had higher PDTI (Additional file 1: Table S1). This indicates that both of these indicators are effective for screening wheat landraces under P-deficiency.

### Molecular markers and population structure

After excluding markers with missing data > 20% and minor allele frequency (MAF) < 0.05, 57,272 polymorphism markers were retained. Based on the ‘Chinese Spring’ physical map v1.0, 21,503 were mapped on A genome, 25,365 were mapped on B genome, and 10,404 markers were mapped D genomes.

Based on the delta-K model [27], using K = 5 had the highest delta-K value. The landraces were divided into five subgroups, of 207, 185, 128, 105, and 82 landraces, respectively. Subgroup 1 comprised all of the landraces from the Xinjiang Winter-Spring Wheat Zone and mainly the landraces from the Northern Winter Wheat Zone and the Yellow and Huai River Valleys Facultative. Subgroup 2 comprised mainly the landraces from the Middle and Low Yangtze Valleys Autumn-Sown Spring Wheat Zone and the Southern Autumn-Sown Spring Wheat Zone. Subgroup 3 comprised mainly the landraces from the Southwestern Autumn-Sown Spring Wheat Zone, and some landraces from the Yellow and Huai River Valleys Facultative Wheat Zone and the Middle and Low Yangtze Valleys Autumn-Sown Spring Wheat Zone. Subgroup 4 mainly included the landraces from the Qinghai-Tibetan Plateau Spring-Winter Wheat Zone and some landraces from the Southwestern Autumn-Sown Spring Wheat Zone, the Northern Spring Wheat Zone, and the Northwestern Spring Wheat Zone. Subgroup 5 was a mixed group, comprising most of the landraces from the Northeastern Spring Wheat Zone, and from parts of other wheat zones.

### Significant loci for seedling traits under AP and NP

Using compressed mixed linear model (CMLM) by TASSEL, a total of 57,272 markers were used to performed a GWAS to detected significant markers for 15 traits under AP and NP. The Bonferroni-corrected threshold (−log_10_^(*p*)^ ≥ 4.76, α = 1) was applied to identify significant markers. Under AP, 61 significant markers, representing 82 MTAs, were detected for 13 traits (PVE: 2.60 to 4.94%) (Additional file 2: Fig. S1 and Additional file 3: Table S2). These 61 significant markers were distributed on chromosomes 1A, 1B, 2A, 2B, 2D, 3A, 4A, 4B, 5A, 5B, 5D, 6A, 6B, 6D, 7A and 7B. Under NP, 22 significant markers (34 MTAs) were detected for 10 traits (PVE 2.66 to 5.40%) (Additional file 2: Fig. S1 and Additional file 3: Table S2). These 22 markers were distributed on chromosomes 2D, 3A, 3D, 4B, 5B, 6A, 6B, 7A and 7B.

A GWAS was also performed using settlement of mixed linear model under progressively exclusive relationship (SUPER) method in genome association and prediction integrated tool (GAPIT) to verify the results of TASSEL. Compared the results from TASSEL, a total of 55 significant markers (81 MTAs) identified by TASSEL were confirmed by SUPER method in GAPIT. These 55 significant markers were used for further analysis. Based on the linkage disequilibrium decay distance, we considered significant markers within a 5.98 Mb region to constitute a single QTL [28]. According to this, 25 QTL were detected under AP, 12 under NP, and 3 under both. Thus, 9 QTL were specific to NP.

Under AP, we identified 25 QTL for 12 traits, distributed on chromosomes 1A, 1B, 2A, 2B, 2D, 3A, 4B, 5A, 5B, 6A, 6D, 7A and 7B **(**Additional file 3: Table S2). For RT, root fresh weight (RFW), DRS, RF, RSA, SDW, TRL, SL, RDW, RL, RV, and TL, 7, 6, 5, 5, 3, 3, 3, 2, 1, 1, 1, and 1 QTL were identified, respectively. Under NP, we identified 12 QTL for 10 traits, distributed on chromosomes 1A, 1B, 2A, 2B, 2D, 3A, 4B, 5A, 5B, 6A, 6D, 7A and 7B **(**Additional file 3: Table S2). For RT, RV, SDW, RDW, RF, RFW, RSA, SFW, SL, TRL, we identified 6, 2, 2, 1, 1, 1, 1, 1, 1 and 1 QTL, respectively.

Three QTL, QTL-2D-3, QTL-4B-2 and QTL-7A-1 were identified under both conditions **(**Additional file 3: Table S2). These three QTL, located on chromosomes 2D, 4B and 7A, were stably expressed under both conditions. Thus, nine QTL were specific to NP conditions. This indicates that these QTL occurred exclusively under P-deficiency stress.

Pleiotropy was revealed by GWAS. Seven QTL were identified for multiple traits. QTL-2D-3 was associated with six traits (RDW, RF, RFW, RSA, RV, and TRL) under NP, and with RF under AP. QTL-4B-2 was associated with six traits, including RF, RFW, RSA, RT, SDW, TRL under AP, and with RT under NP. This finding is supported by Pearson’s correlation analysis, with the correlation coefficients among these six traits ranging from 0.578 (between SDW and TRL) to 0.976 (between RSA and TRL).

## Discussion

As an important plant organ for P uptake and utilization, the root system has been used in screening P-deficiency-tolerant wheat genotypes [29]. The seedling stage often determines the traits of the mature stage, and the traits of the two stages are closely related. The QTL associated with N-absorption rate detected in a field experiment, and those associated with seedling character in greenhouse hydroponic culture, were co-located [30]. Further, the QTL of root hair on Chr. 2A and 6A were also associated with yield-related traits [31]. These results indicate that nutrient absorption and utilization during the seedling stage can affect the phenotype in the mature stage. The traits showed significant variation in their responses to P deficiency. Most showed moderate to high heritability (Table 2), revealing their potential for analysis in our next GWAS. Most of the traits were significantly correlated, and the six root traits were moderately to highly correlated. High correlations between wheat-root seedling traits have also been reported in previous studies [32–35]. Those studies revealed that seedling root traits are inherited together, and that it is difficult to independently select for one of these traits.

Here, the first three PCs explained more than 80% phenotypic variation and included all tested traits (Table 4). The first PC mostly reflected the contribution of 12 traits (with the exception of DRS, FRS and RD). The second and third PCs reflected the contributions of root–shoot ratio and RD, respectively. We found that increasing root–shoot ratio led to reduction in aboveground biomass. DRS, FRS, and RD were higher under NP than under AP, whereas the other traits were lower under NP. Increases in the root–shoot ratio in response to stress have been reported previously [28, 36]. Plants can increase their root–shoot ratio to promote P uptake and utilization in response to low-P stress [37]. ‘Chinese Spring’ was identified as sensitive to P deficiency (*S* = − 1.16), consistent with previous studies [19, 26, 38]. It indicated that using S value was a reliable method to screen wheat tolerant genotypes. Finally, ten extremely sensitive and ten extremely tolerant landraces were selected for further genetic analysis and for breeding tolerant cultivars (Additional file 1: Table S1).

The SUPER method is a power GWAS for identifying QTL as it extracts a small subset of single-nucleotide polymorphisms (SNPs) and use them in FaST-LMM [39]. This method can retain the computational advantages of FaST-LMM and increase statistical power [39]. A total of 81 of 116 (69.83%) MTAs by CMLM were confirmed by SUPER method. Comparing with CMLM, the number of MTAs identified using SUPER were greatly increased (Additional file 2: Fig. S1). However, quantile-quantile (Q-Q) plots from different algorithms revealed that CMLM was fitted better than SUPER (Additional file 2: Fig. S1). Previous studies revealed the similar results [40, 41].

Pleiotropy was identified in seven QTL. QTL for different highly correlated traits may be located in the same chromosomal regions [42]. We found that all traits except DRS, FRS, and RD, were significantly positively correlated under both conditions. The six root morphological traits were moderately to highly correlated (Table 2). Pleiotropy has been observed in previous studies [19, 32, 33, 43]. QTL-2D-3, located on Chr. 2D at 186.65 Mb, was associated with RDW, RF, RFW, RT, TRL, RSA, and RV under NP, and with RF under AP. In a previous study, QTL associated with P-use efficiency were identified on Chr. 2D [44]. We found that QTL-7A-1, located on Chr. 7A at 52.03 Mb, was associated with RT and RFW under AP, and with RT under NP. This indicates that QTL-7A-1 may regulate and control RT under both AP and NP, and root development under AP. Previous studies have found that Chr. 7A may play important roles in response to P deficiency [44, 45].

## Conclusions

We evaluated 15 traits in 707 Chinese wheat landraces, under application of P or non-application of P. Ten extremely tolerant and ten extremely sensitive accessions were selected as germplasm materials for further study. In total, 25 and 12 QTL were identified under AP and NP, respectively, while nine of 12 were specific to NP. In total, seven QTL showed pleiotropy, and several QTL had been previously identified. Our findings provide new insight into the genetic analysis of P-deficiency-tolerance, and will be helpful for breeding P-deficiency-tolerant cultivars.

## Methods

### Plant Germplasm

The 707 accessions used in this study were from a core collection of wheat landraces [46] originating from ten agro-ecological zones in China (Additional file 4**:** Table S3).

### Glasshouse experiments and phenotypic data collection

All landraces were grown hydroponically in a greenhouse at the Triticeae Research Institute, Sichuan Agricultural University. A completely randomized design, each with three replications, was used in this study. The greenhouse environment, hydroponic system, and phenotypic date collection were as previously described [26, 47, 48]. Briefly, the AP and NP treatments contained the modified from Hoagland’s nutrient solution [26, 47–49] with and without NH_4_H_2_PO_4_ (1 mmol/L), respectively. Two sets of seedlings were grown for 3 day under AP, and were then grown for 12 day under AP and NP, respectively. Solutions were replaced every 4 day. After 12 day of growth, phenotypic data of all traits were gathered as our previous described method [28].

### Phenotypic data analysis

To eliminate environmental effects, the best linear unbiased prediction (BLUP) values across three repetitions were conducted using the MIXED procedure in SAS [50]. The BLUP values for each trait were used to determine descriptive statistics, for ANOVA testing, and to obtain the *H′* and Pearson correlation coefficients, using IBM SPSS Statistics for Windows 20.0 (IBM Corp., Chicago, IL, USA). The *H*^*2*^ was calculated using the formula *H*^2^ = *V*_*G*_/(V_*G*_ + *V*_*E*_/*r*), where *V*_*G*_ is the genotypic variance, *V*_*E*_ is the environment variance, and r is the number of replications [51]. To screen for P-deficiency-tolerance, we used a weighting method to acquire the *S* value of each landrace genotype. The *S* value was calculated using the following $S={\sum}_{i=1}^k ri Yi/{\sum}_{i=1}^k ri$ [26].

### Genotyping and population structure analyses

Genomic DNA was extracted using the CTAB method. Genotyping-by-sequencing libraries (96-plex) were constructed via the two-enzyme method [52], and sequenced on the Illumina HiSeq 2500 system. SNP calling was done using the Tassel pipeline [53]. The physical distances of SNP were based on the Chinese Spring reference sequence v1.0 [54]. SNPs without physical distances were removed. The linkage disequilibrium K-number neighbor imputation method was used for imputation accuracies [55]. Finally, 18,594 SNPs were retained with missing data ≤20% and MAF ≥ 0.05. Besides, 38,678 DArT-seq markers from our previous study were also used for GWAS [46].

Population structure was evaluated using STRUCTURE 2.3.4, implementing model-based Bayesian cluster analysis [56]. Based on the admixture model, population genetic clusters of K = 1 to K = 10 were estimated with 10,000 replicates for burn-in and 10,000 replicates for MCMC. Five runs were set for each K. The optimal K value was determined using STRUCTURE HARVESTER [57] implementing the Evanno method [27]. The optimal alignment of the five repeated runs was determined using CLUMPP [58].

### GWAS

In total, 57,272 markers (18,594 SNP and 38,678 DArT-seq markers) were used to perform GWAS, using TASSEL 5.2.60 [59]. A compressed mixed liner model were used to detect marker–trait associations [60, 61] with the *Q* matrix and kinship matrix as covariates by TASSEL v5.2.60. Furthermore, the significant markers identified from TASSEL v5.2.60 were confirmed using SUPER method [39] in GAPIT [62] implemented in the R 3.6.3 [63]. Significant markers for traits identified by both TASSEL and GAPIT were used for further analysis. Based on the Bonferroni-corrected *p*-value threshold α = 1 [62–64], the threshold value of significant markers was set as -log10(p) = 4.76. Manhattan and Q-Q plots of GWAS results were drawn using R 3.6.3 [63] as our previous studies [28, 46].

## Supplementary information

**Additional file 1: Table S1.** Top ten genotypes with extremely tolerance and bottommost ten genotypes with extremely sensitivity selected from 707 Chinese wheat landraces based on phosphorus deficiency tolerance index (PDTI) and synthesis value (*S* value).

**Additional file 2: Fig. S1.** Manhattan plots and quantile-quantile (Q-Q) plots for 15 seedling traits under application of phosphorus (AP) and non-application of phosphorus (NP).

**Additional file 3: Table S2.** List of the significant markers for all seedling traits in two conditions by TASSEL and GAPIT. Different QTL are distinguished by yellow and green color.

**Additional file 4: Table S3.** Information of the 707 wheat accessions assessed in the present study.

## Data Availability

The datasets used and/or analysed during the current study are available from the corresponding author on reasonable request. SNPs data used in this study is availability in ENA at https://www.ebi.ac.uk/ena/browser/view/ERZ1309082 (Analysis Accession: ERZ1309082).

## Abbreviations

AP: Application of phosphorus
BLUP: Best linear unbiased prediction
CMLM: Compressed mixed liner model
DArT-seq: Diversity arrays technology sequencing
DRS: Dry root–shoot ratio
FRS: Fresh root–shoot ratio
GAPIT: Genome association and prediction integrated tool
GWAS: Genome-wide association studies
*H′*: Shannon–Weaver diversity index
*H*^*2*^: Broad-sense heritability
MAF: Minor allele frequency
MTAs: Marker–trait associations
NP: Non-application of phosphorus
P: Phosphorus
PC: Principal component
PDTI: P-deficiency tolerance index
PVE: Phenotypic variation explained
Q-Q plots: Quantile-quantile plots
QTL: Quantitative trait loci
RD: Root diameter
RDW: Root dry weight
RF: Root forks
RFW: Root fresh weight
RL: Root length
RSA: Root surface area
RT: Root tips
RV: Root volume
SDW: Shoot dry weight
SFW: Shoot fresh weight
SL: Shoot length
SNPs: Single-nucleotide polymorphisms
SUPER: Settlement of mixed linear model under progressively exclusive relationship
S value: Synthesis value
TL: Total length of shoot and root
TRL: Total root length
