# Supplementary material for: Phenotypic and genetic variation in phosphorus-deficiency-tolerance traits in Chinese wheat landraces
Source: BMC Plant Biol. 2020 Jul 13;20:330. doi: 10.1186/s12870-020-02492-3 (PMC7359013; doi:10.1186/s12870-020-02492-3)
Supplement: Supplementary file 2 — Additional file 2: Fig. S1. Manhattan plots and quantile-quantile (Q-Q) plots for 15 seedling traits under application of phosphorus (AP) and non-application of phosphorus (NP). [file 12870_2020_2492_MOESM2_ESM.pdf]

Figure S1 Manhattan plots and quantile-quantile (Q-Q) plots for 15 seedling traits under application of phosphorus (AP) and non-application of phosphorus (NP).
(a) Manhattan plots under AP using compressed mixed linear model (CMLM) in TASSEL.
(b) Manhattan plots under NP using CMLM in TASSEL.
(c) Manhattan plots under AP using settlement of MLM under progressively exclusive relationship (SUPER) in the genome association and prediction integrated tool (GAPIT).
(d) Manhattan plots under NP using SUPER method in GAPIT.
(e) Comparison of Q-Q plots between using CMLM in TASSEL and SUPER method in GAPIT under AP.
(f) Comparison of Q-Q plots between using CMLM in TASSEL and SUPER method in GAPIT under NP.
The darkgoldenrod plots indicate using CMLM in TASSEL, whereas darkgreen histograms indicate using SUPER method in GAPIT. The solid black line indicated the expected values.

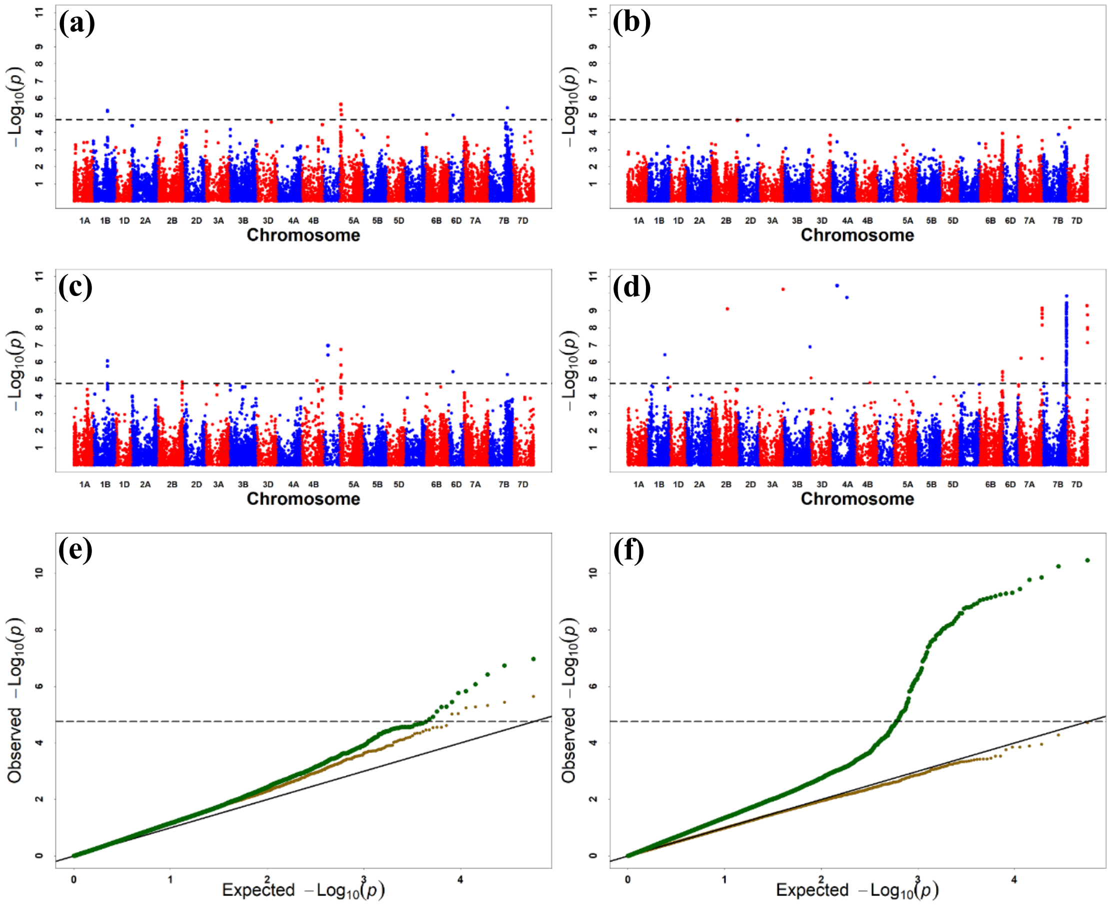

Dry root-shoot ratio (DRS)

(a) Manhattan plots for DRS under AP conditions using CMLM in TASSEL.

(b) Manhattan plots for DRS under NP conditions using CMLM in TASSEL.

(c) Manhattan plots for DRS under AP conditions using SUPER method in GAPIT.

(d) Manhattan plots for DRS under NP conditions using SUPER method in GAPIT.

(e) Q-Q plots for DRS under AP using CMLM in TASSEL and SUPER method in GAPIT.

(f) Q-Q plots for DRS under NP using CMLM in TASSEL and SUPER method in GAPIT.

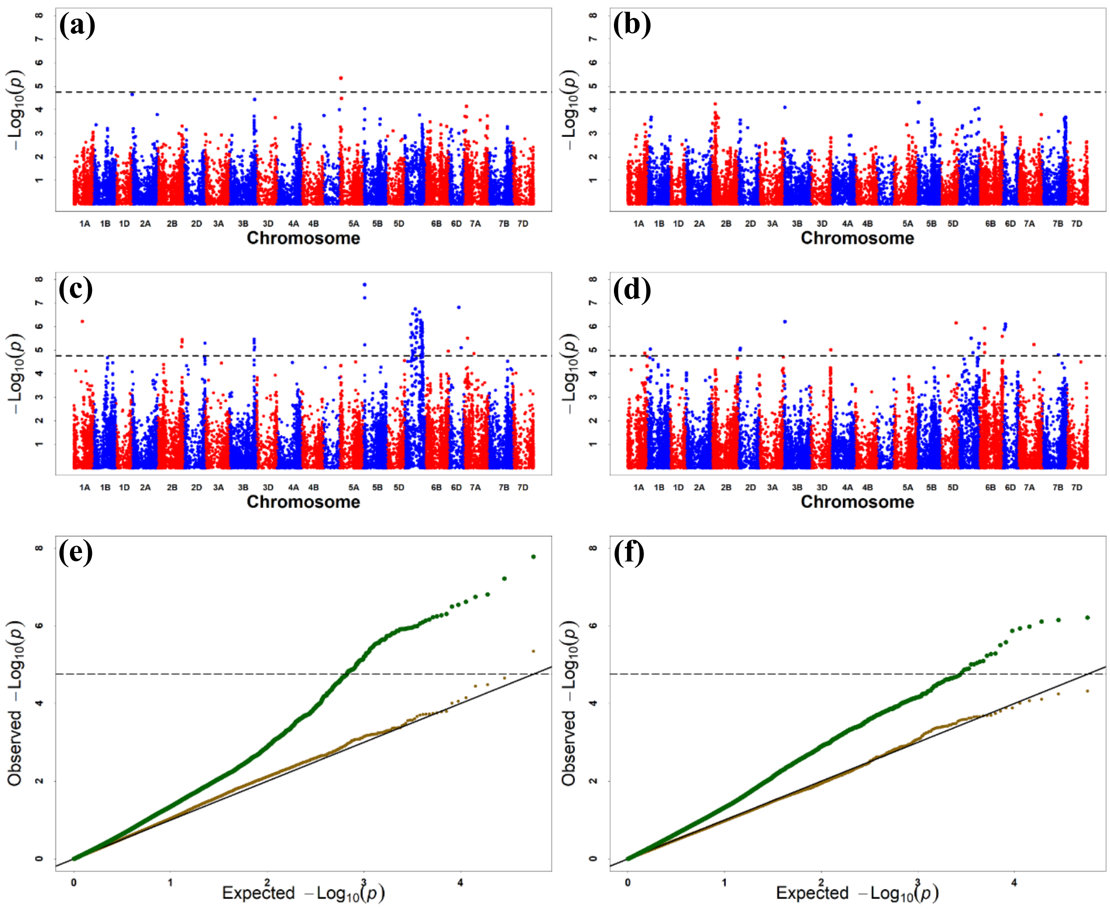

Fresh root-shoot ratio (FRS)

(a) Manhattan plots for FRS under AP conditions using CMLM in TASSEL.

(b) Manhattan plots for FRS under NP conditions using CMLM in TASSEL.

(c) Manhattan plots for FRS under AP conditions using SUPER method in GAPIT.

(d) Manhattan plots for FRS under NP conditions using SUPER method in GAPIT.

(e) Q-Q plots for FRS under AP using CMLM in TASSEL and SUPER method in GAPIT.

(f) Q-Q plots for FRS under NP using CMLM in TASSEL and SUPER method in GAPIT.

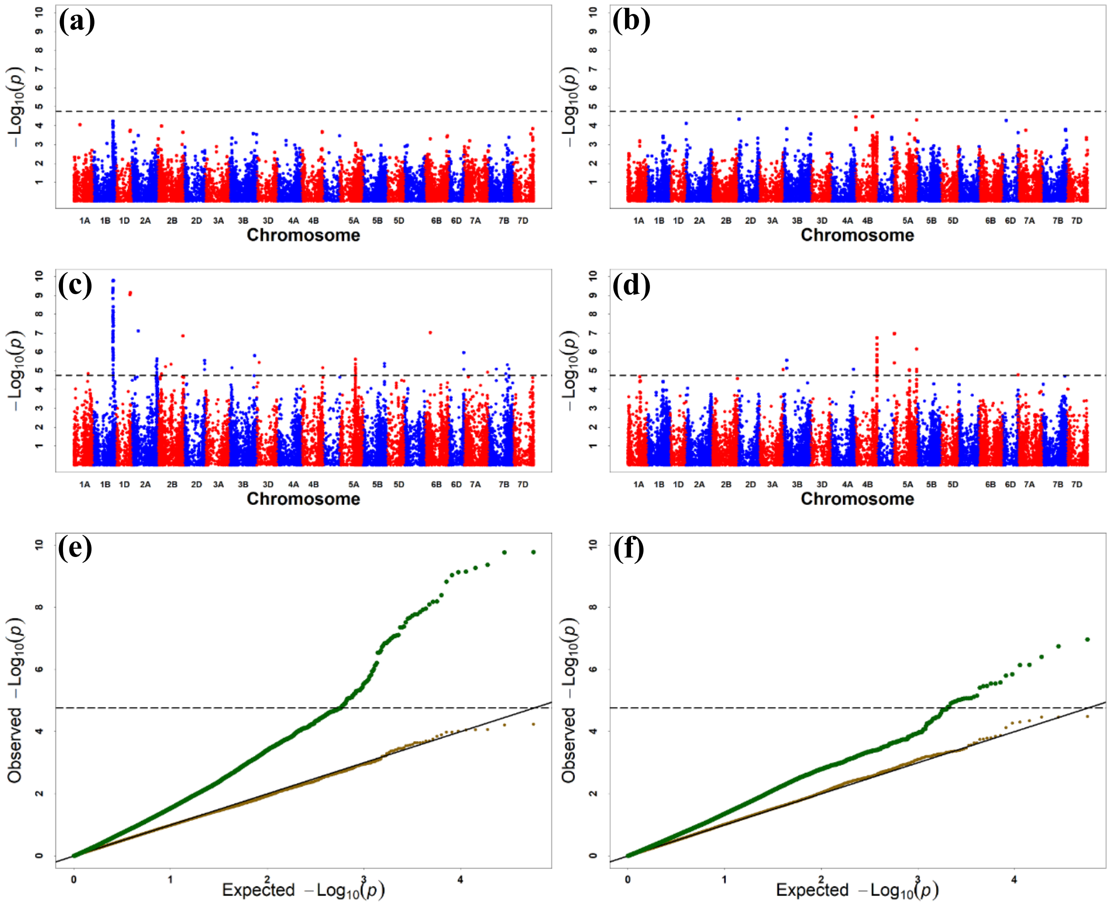

Root diameter (RD)

(a) Manhattan plots for RD under AP conditions using CMLM in TASSEL.

(b) Manhattan plots for RD under NP conditions using CMLM in TASSEL.

(c) Manhattan plots for RD under AP conditions using SUPER method in GAPIT.

(d) Manhattan plots for RD under NP conditions using SUPER method in GAPIT.

(e) Q-Q plots for RD under AP using CMLM in TASSEL and SUPER method in GAPIT.

(f) Q-Q plots for RD under NP using CMLM in TASSEL and SUPER method in GAPIT.

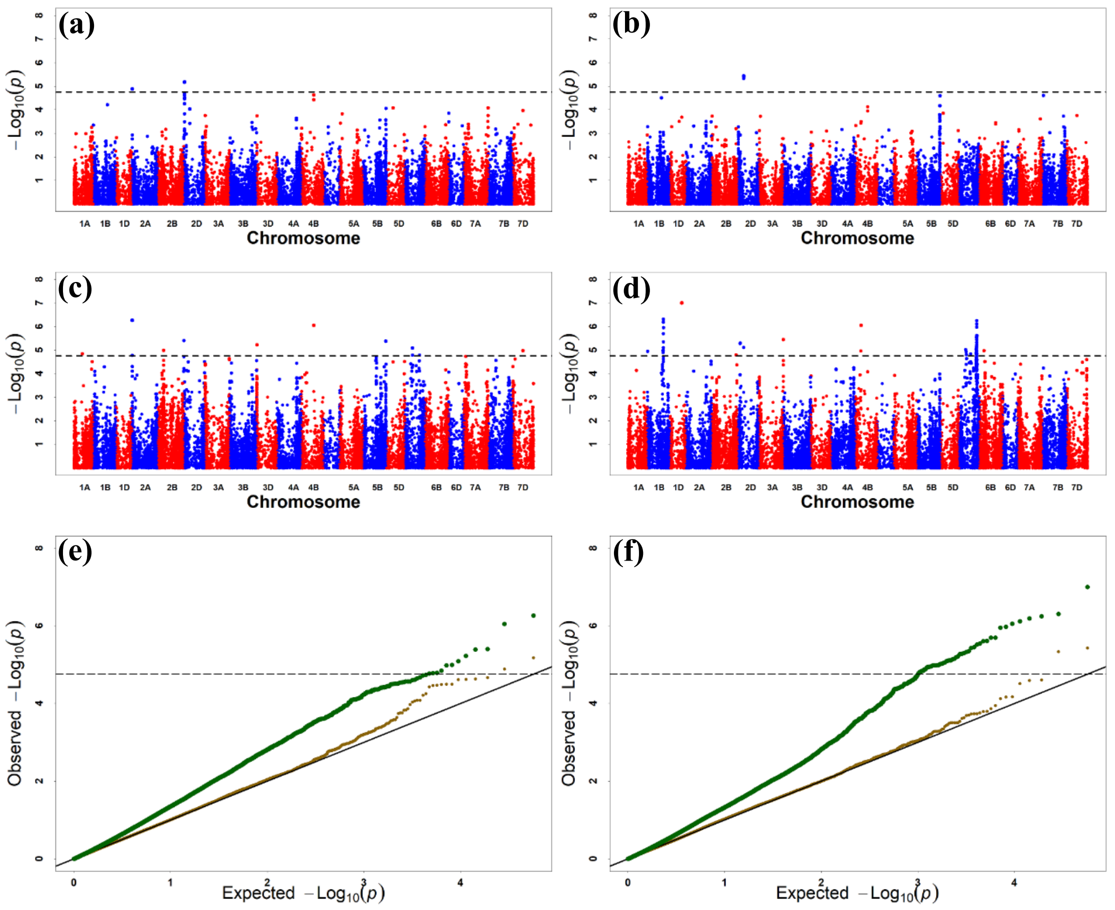

Root dry weight (RDW)

(a) Manhattan plots for RDW under AP conditions using CMLM in TASSEL.

(b) Manhattan plots for RDW under NP conditions using CMLM in TASSEL.

(c) Manhattan plots for RDW under AP conditions using SUPER method in GAPIT.

(d) Manhattan plots for RDW under NP conditions using SUPER method in GAPIT.

(e) Q-Q plots for RDW under AP using CMLM in TASSEL and SUPER method in GAPIT.

(f) Q-Q plots for RDW under NP using CMLM in TASSEL and SUPER method in GAPIT.

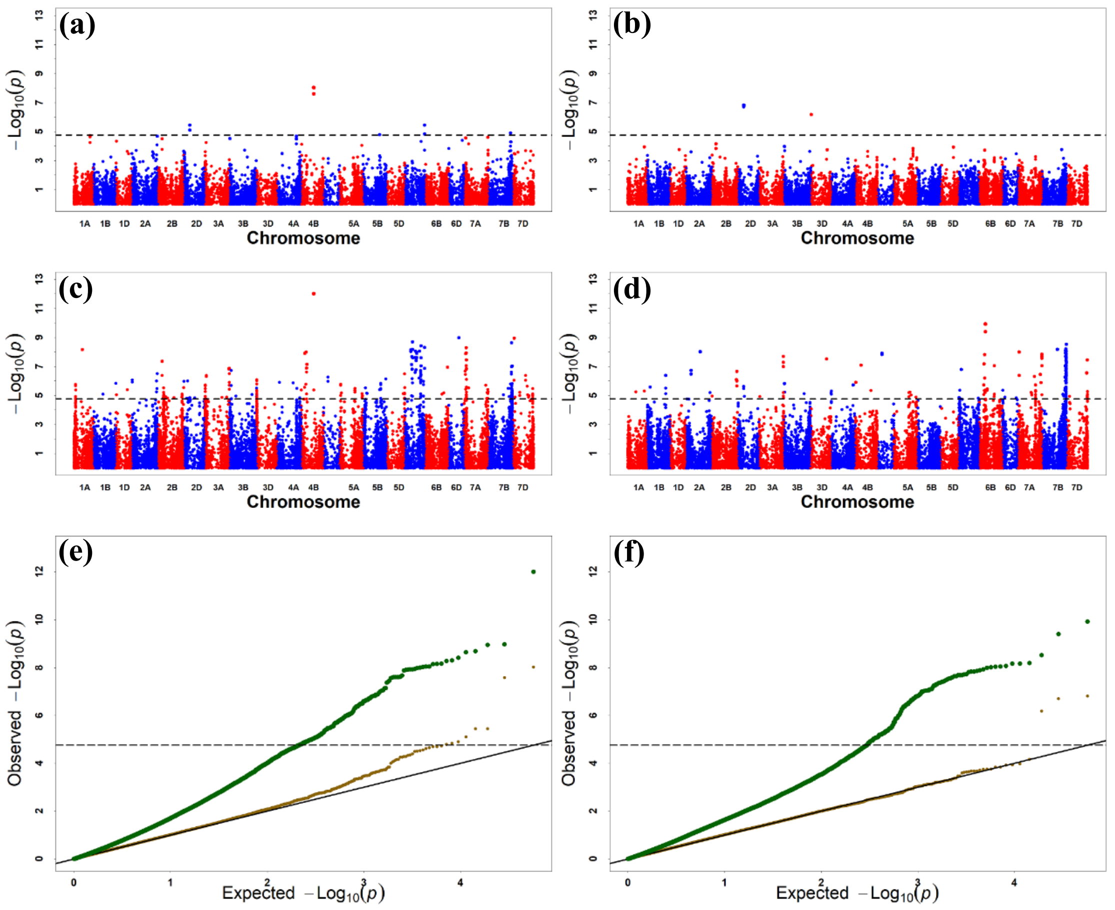

Root forks (RF)

(a) Manhattan plots for RF under AP conditions using CMLM in TASSEL.

(b) Manhattan plots for RF under NP conditions using CMLM in TASSEL.

(c) Manhattan plots for RF under AP conditions using SUPER method in GAPIT.

(d) Manhattan plots for RF under NP conditions using SUPER method in GAPIT.

(e) Q-Q plots for RF under AP using CMLM in TASSEL and SUPER method in GAPIT.

(f) Q-Q plots for RF under NP using CMLM in TASSEL and SUPER method in GAPIT.

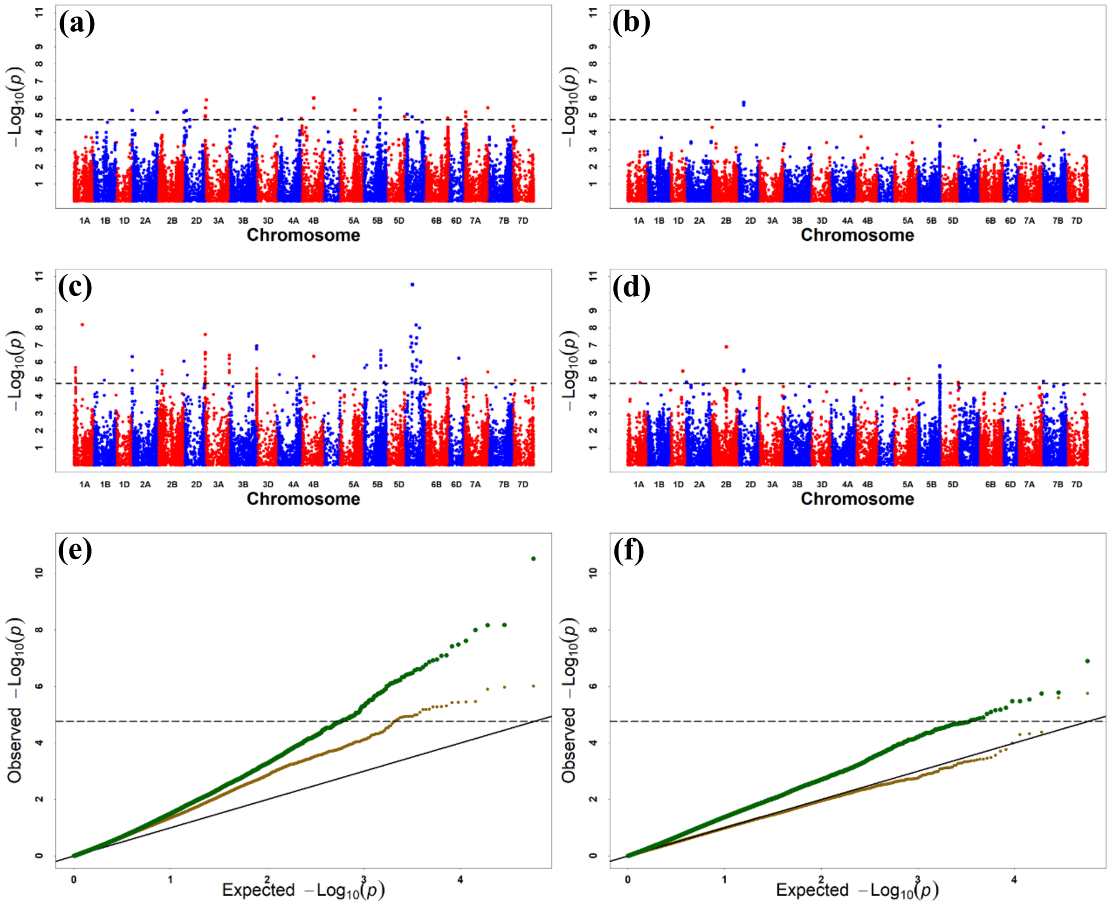

Root fresh weight (RFW)

(a) Manhattan plots for RFW under AP conditions using CMLM in TASSEL.

(b) Manhattan plots for RFW under NP conditions using CMLM in TASSEL.

(c) Manhattan plots for RFW under AP conditions using SUPER method in GAPIT.

(d) Manhattan plots for RFW under NP conditions using SUPER method in GAPIT.

(e) Q-Q plots for RFW under AP using CMLM in TASSEL and SUPER method in GAPIT.

(f) Q-Q plots for RFW under NP using CMLM in TASSEL and SUPER method in GAPIT.

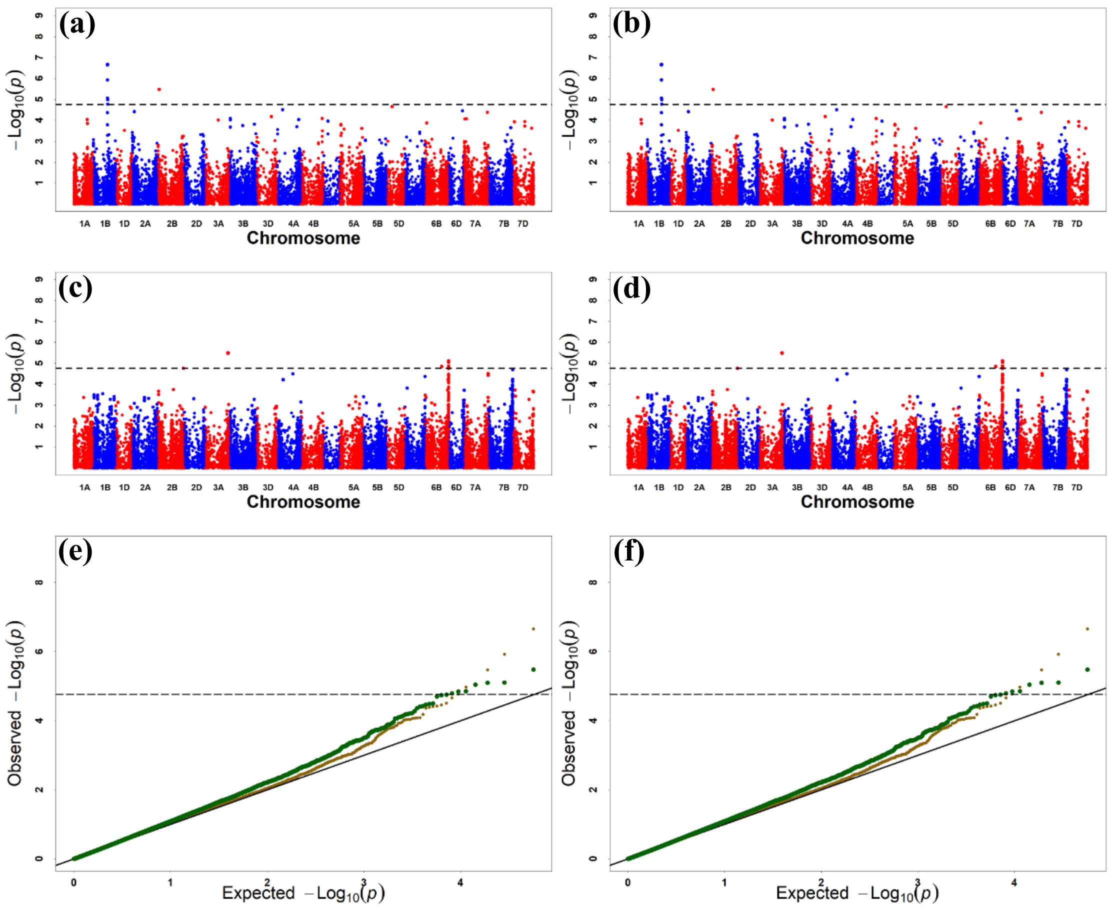

Root length (RL)

(a) Manhattan plots for RL under AP conditions using CMLM in TASSEL.

(b) Manhattan plots for RL under NP conditions using CMLM in TASSEL.

(c) Manhattan plots for RL under AP conditions using SUPER method in GAPIT.

(d) Manhattan plots for RL under NP conditions using SUPER method in GAPIT.

(e) Q-Q plots for RL under AP using CMLM in TASSEL and SUPER method in GAPIT.

(f) Q-Q plots for RL under NP using CMLM in TASSEL and SUPER method in GAPIT.

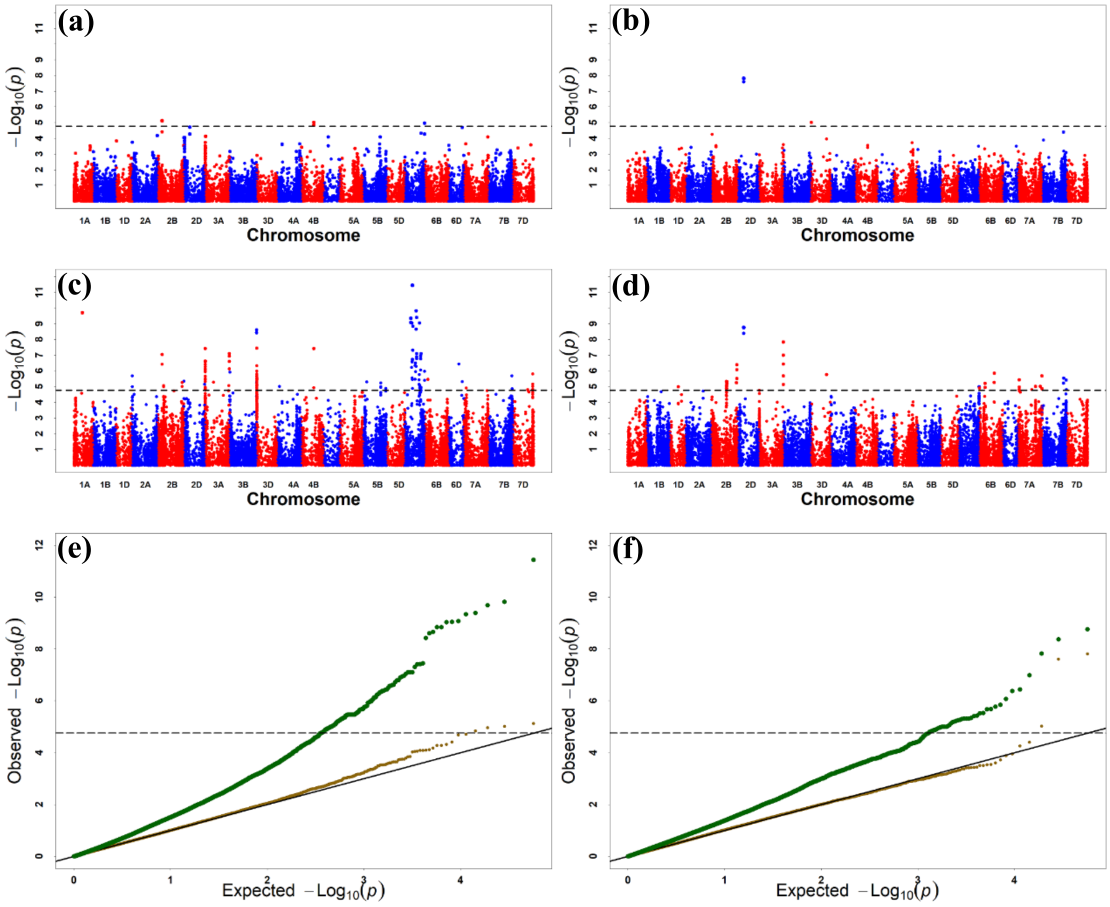

Root surface area (RSA)

(a) Manhattan plots for RSA under AP conditions using CMLM in TASSEL.

(b) Manhattan plots for RSA under NP conditions using CMLM in TASSEL.

(c) Manhattan plots for RSA under AP conditions using SUPER method in GAPIT.

(d) Manhattan plots for RSA under NP conditions using SUPER method in GAPIT.

(e) Q-Q plots for RSA under AP using CMLM in TASSEL and SUPER method in GAPIT.

(f) Q-Q plots for RSA under NP using CMLM in TASSEL and SUPER method in GAPIT.

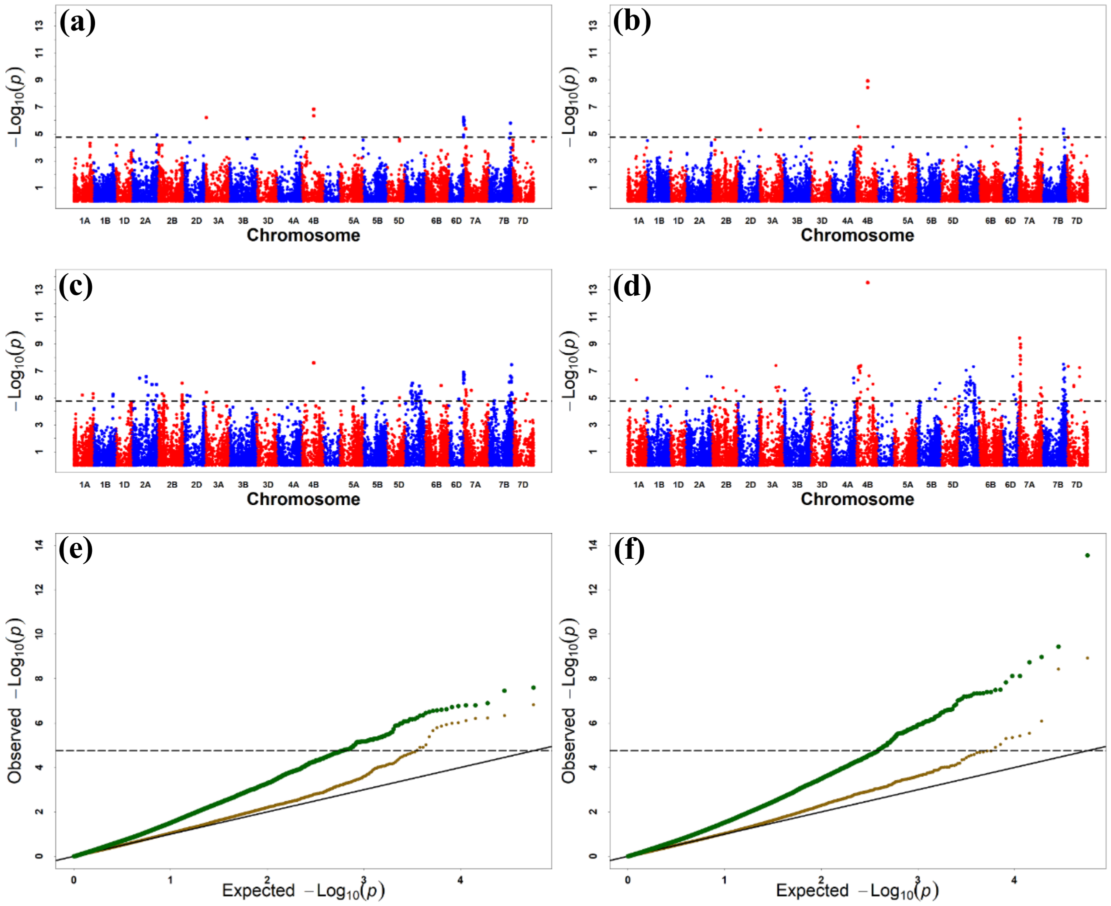

Root tips (RT)

(a) Manhattan plots for RT under AP conditions using CMLM in TASSEL.

(b) Manhattan plots for RT under NP conditions using CMLM in TASSEL.

(c) Manhattan plots for RT under AP conditions using SUPER method in GAPIT.

(d) Manhattan plots for RT under NP conditions using SUPER method in GAPIT.

(e) Q-Q plots for RT under AP using CMLM in TASSEL and SUPER method in GAPIT.

(f) Q-Q plots for RT under NP using CMLM in TASSEL and SUPER method in GAPIT.

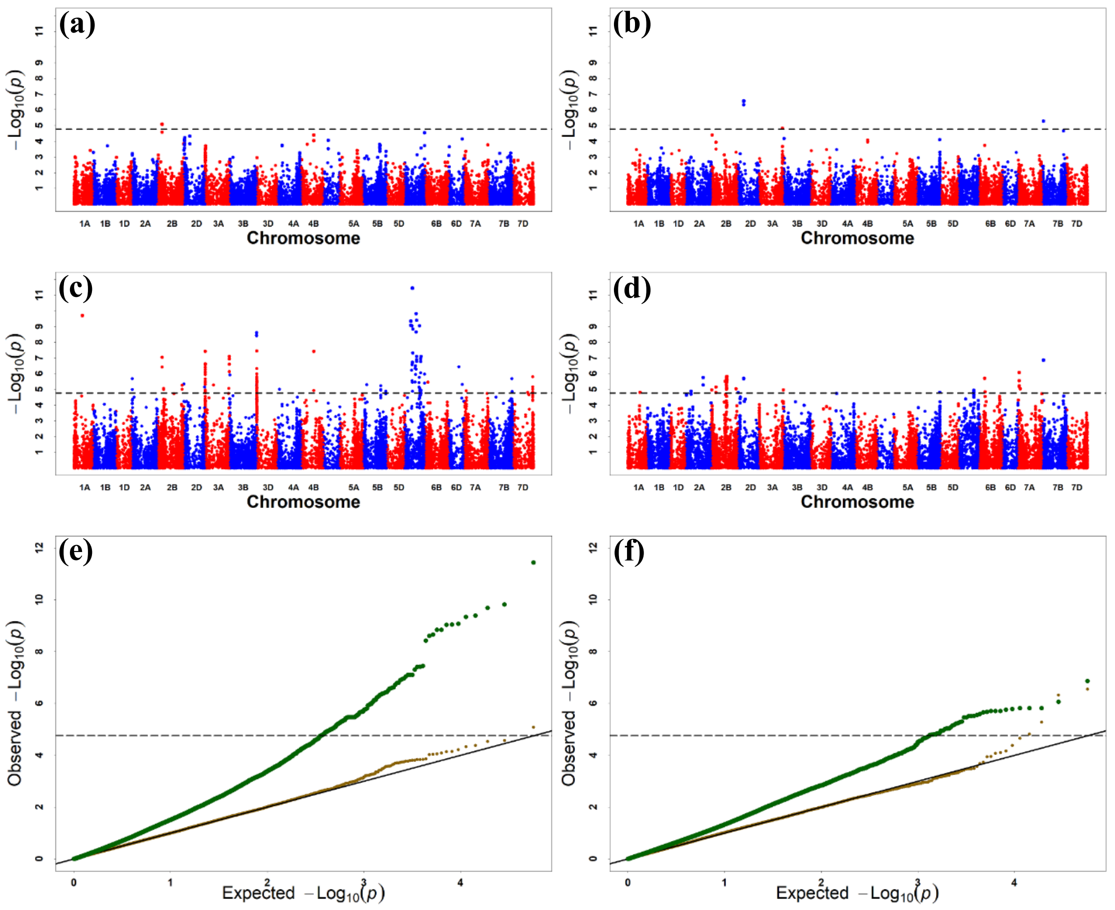

Root volume (RV)
(a) Manhattan plots for RV under AP conditions using CMLM in TASSEL.
(b) Manhattan plots for RV under NP conditions using CMLM in TASSEL.
(c) Manhattan plots for RV under AP conditions using SUPER method in GAPIT.
(d) Manhattan plots for RV under NP conditions using SUPER method in GAPIT.
(e) Q-Q plots for RV under AP using CMLM in TASSEL and SUPER method in GAPIT.
(f) Q-Q plots for RV under NP using CMLM in TASSEL and SUPER method in GAPIT.

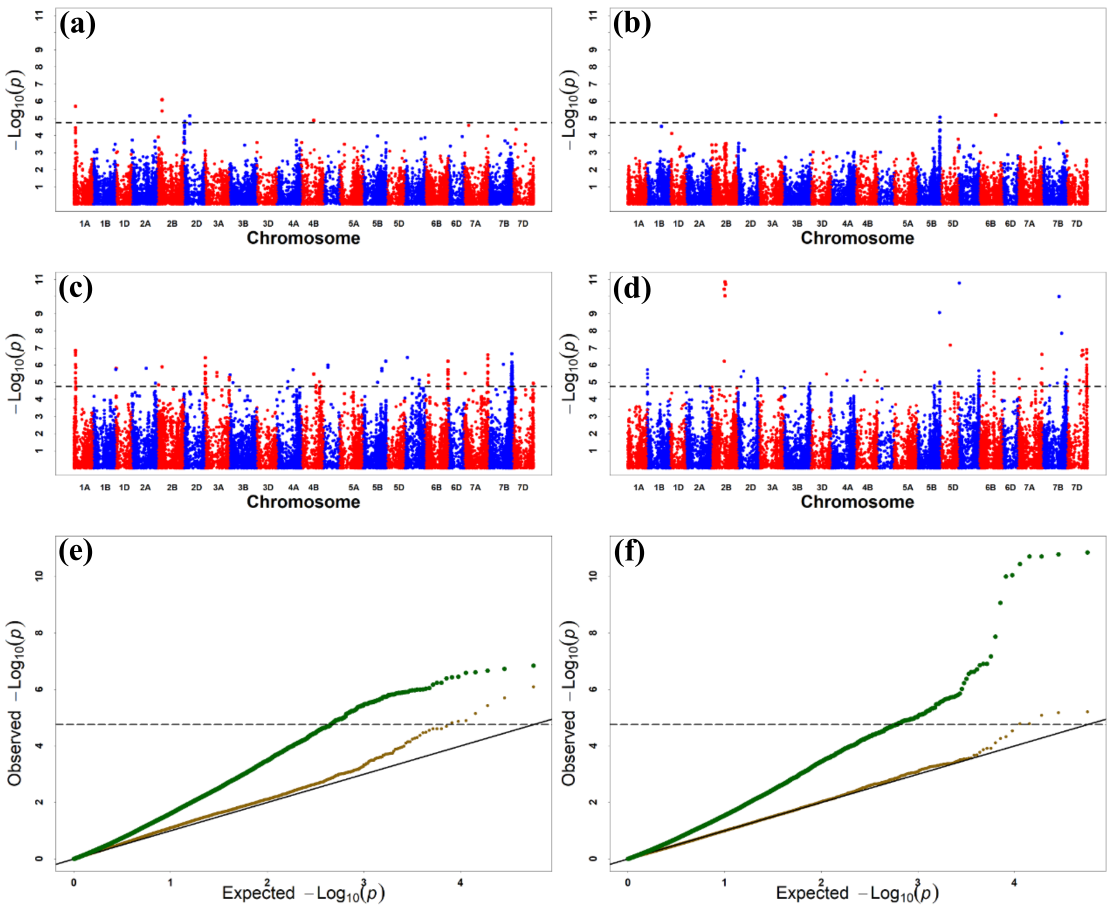

Shoot dry weight (SDW)

(a) Manhattan plots for SDW under AP conditions using CMLM in TASSEL.

(b) Manhattan plots for SDW under NP conditions using CMLM in TASSEL.

(c) Manhattan plots for SDW under AP conditions using SUPER method in GAPIT.

(d) Manhattan plots for SDW under NP conditions using SUPER method in GAPIT.

(e) Q-Q plots for SDW under AP using CMLM in TASSEL and SUPER method in GAPIT.

(f) Q-Q plots for SDW under NP using CMLM in TASSEL and SUPER method in GAPIT.

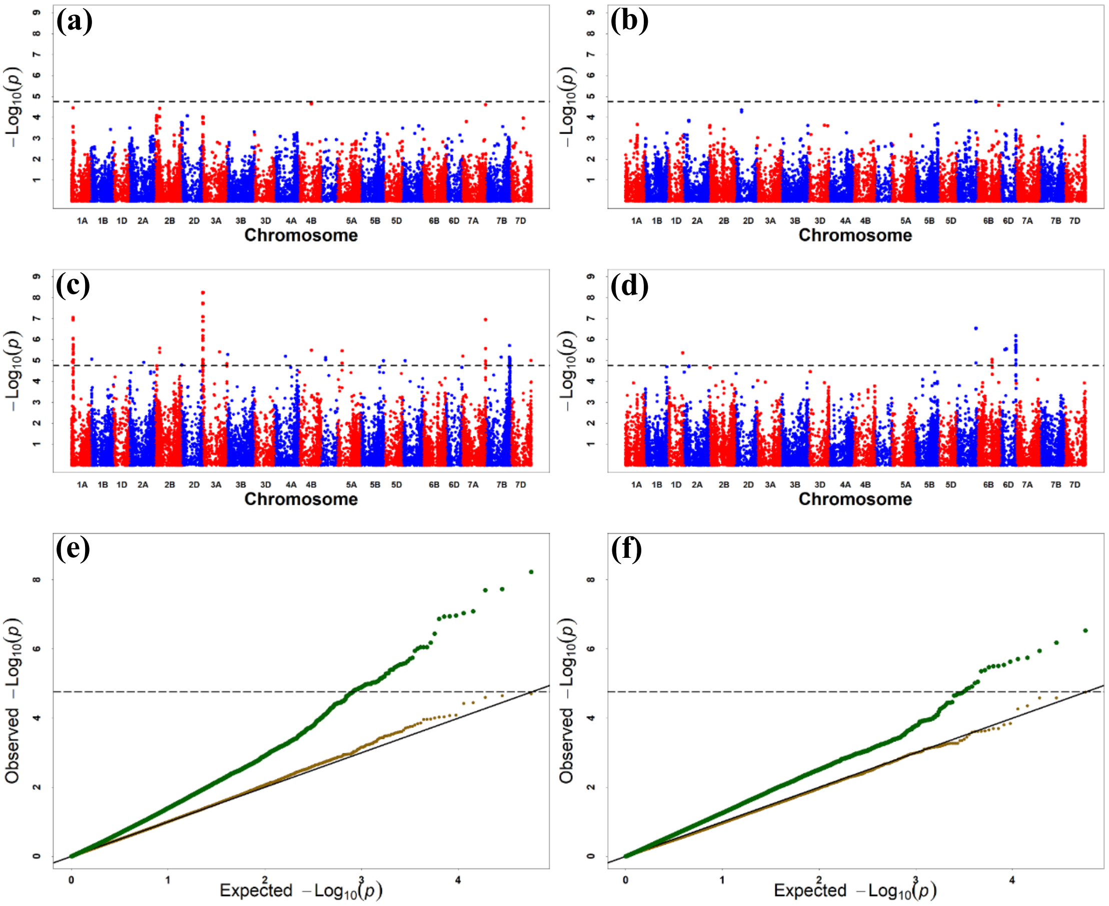

Shoot fresh weight (SFW)

(a) Manhattan plots for SFW under AP conditions using CMLM in TASSEL.

(b) Manhattan plots for SFW under NP conditions using CMLM in TASSEL.

(c) Manhattan plots for SFW under AP conditions using SUPER method in GAPIT.

(d) Manhattan plots for SFW under NP conditions using SUPER method in GAPIT.

(e) Q-Q plots for SFW under AP using CMLM in TASSEL and SUPER method in GAPIT.

(f) Q-Q plots for SFW under NP using CMLM in TASSEL and SUPER method in GAPIT.

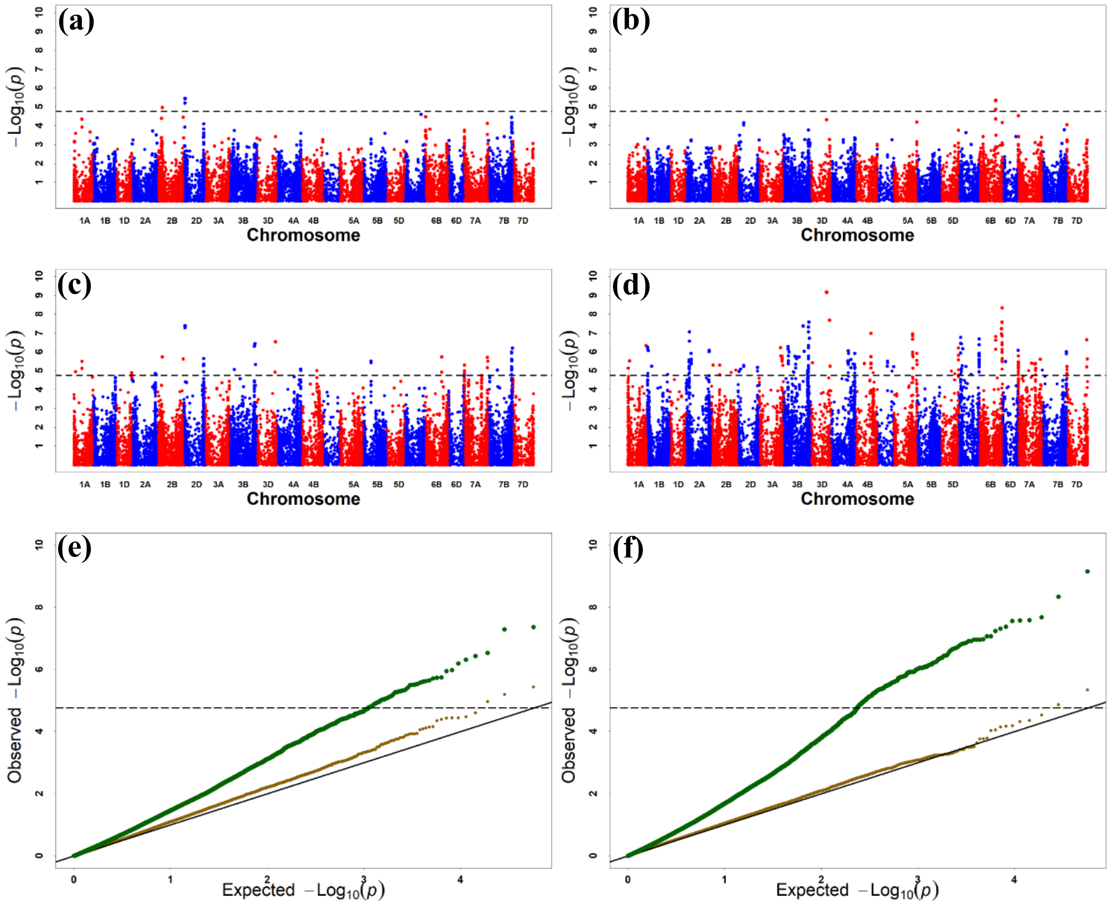

Shoot length (SL)

(a) Manhattan plots for SL under AP conditions using CMLM in TASSEL.

(b) Manhattan plots for SL under NP conditions using CMLM in TASSEL.

(c) Manhattan plots for SL under AP conditions using SUPER method in GAPIT.

(d) Manhattan plots for SL under NP conditions using SUPER method in GAPIT.

(e) Q-Q plots for SL under AP using CMLM in TASSEL and SUPER method in GAPIT.

(f) Q-Q plots for SL under NP using CMLM in TASSEL and SUPER method in GAPIT.

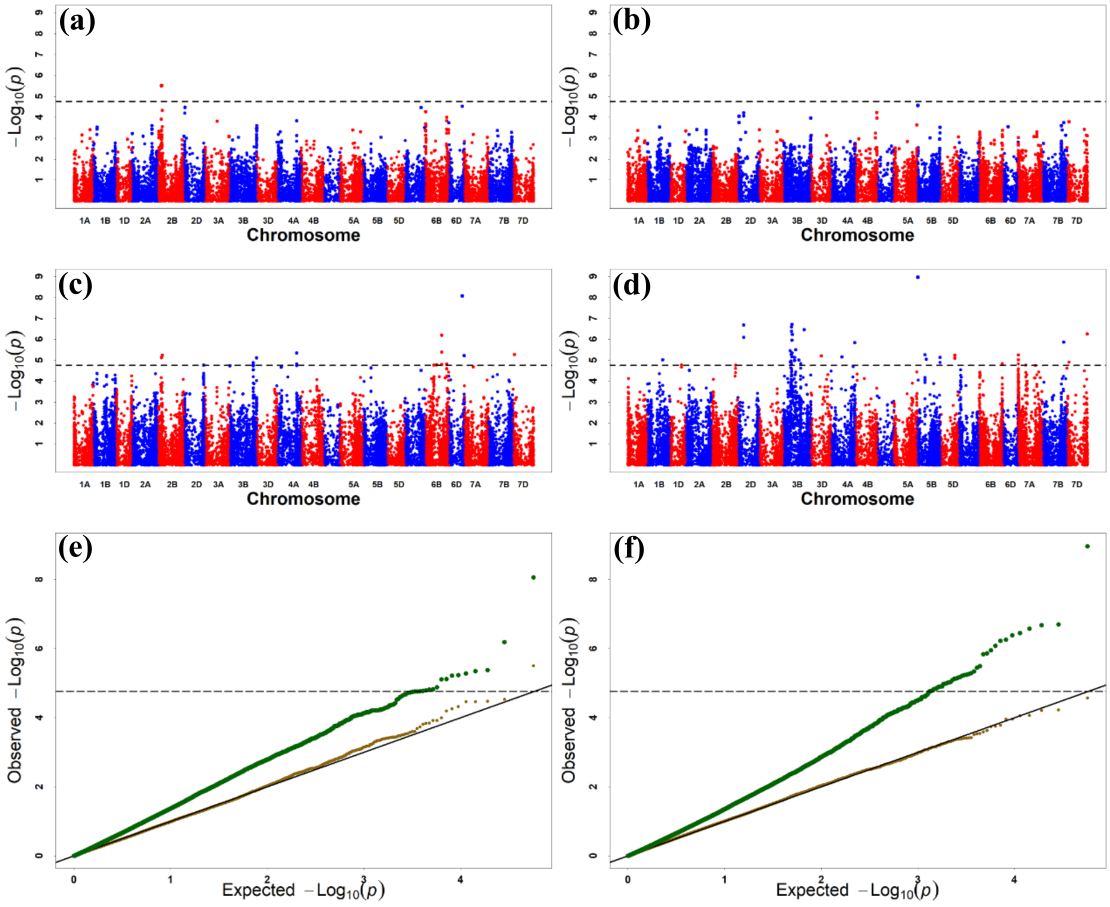

Total length of shoot and root (TL)

(a) Manhattan plots for TL under AP conditions using CMLM in TASSEL.

(b) Manhattan plots for TL under NP conditions using CMLM in TASSEL.

(c) Manhattan plots for TL under AP conditions using SUPER method in GAPIT.

(d) Manhattan plots for TL under NP conditions using SUPER method in GAPIT.

(e) Q-Q plots for TL under AP using CMLM in TASSEL and SUPER method in GAPIT.

(f) Q-Q plots for TL under NP using CMLM in TASSEL and SUPER method in GAPIT.

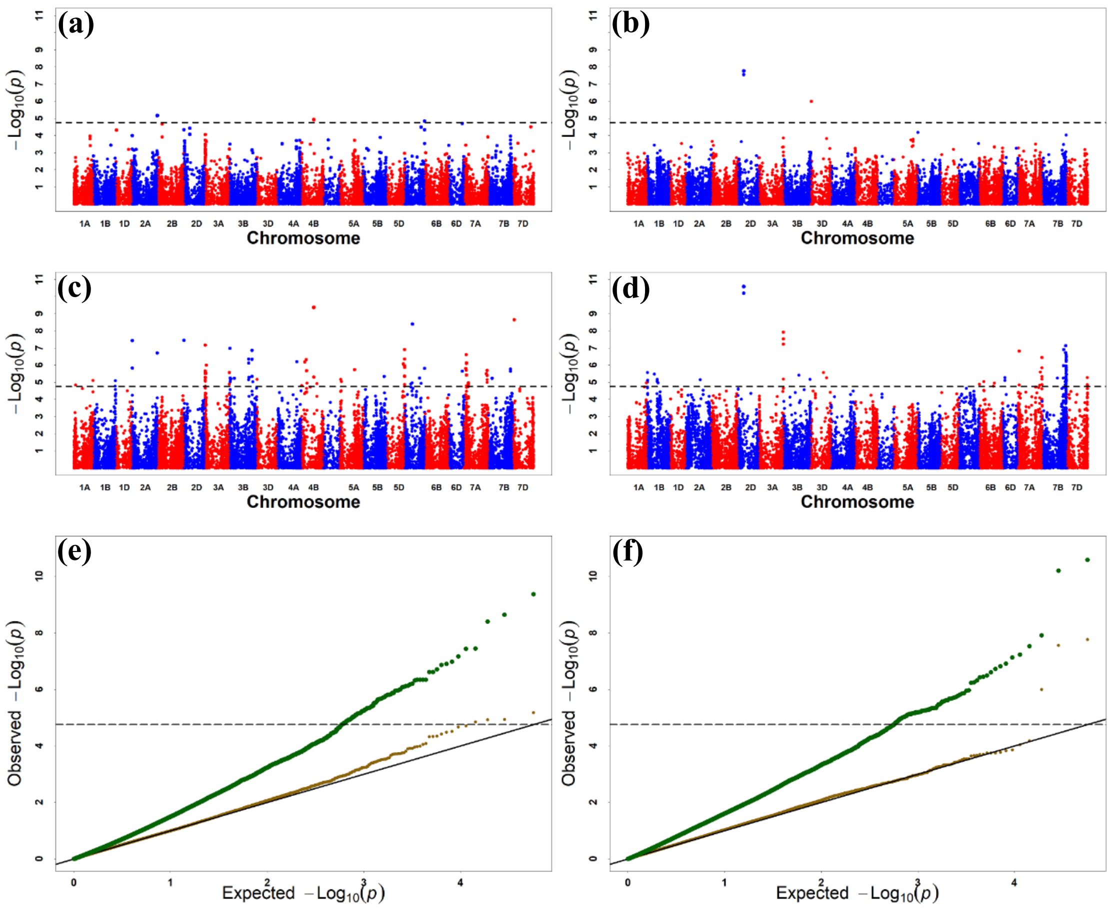

Total root length (TRL)

(a) Manhattan plots for TRL under AP conditions using CMLM in TASSEL.

(b) Manhattan plots for TRL under NP conditions using CMLM in TASSEL.

(c) Manhattan plots for TRL under AP conditions using SUPER method in GAPIT.

(d) Manhattan plots for TRL under NP conditions using SUPER method in GAPIT.

(e) Q-Q plots for TRL under AP using CMLM in TASSEL and SUPER method in GAPIT.

(f) Q-Q plots for TRL under NP using CMLM in TASSEL and SUPER method in GAPIT.
